# The Effect of Dietary Concentrations of Digestible Lysine and Sulphur Amino Acids on The Productive Performance and Egg Quality Traits in Aged Laying Hens

**DOI:** 10.1002/vms3.70789

**Published:** 2026-01-14

**Authors:** Ahmad Hassanabadi, Elnaz Fallah Moghadam, Heydar Zarghi

**Affiliations:** ^1^ Department of Animal Science Faculty of Agriculture Ferdowsi University of Mashhad Mashhad Iran

**Keywords:** egg quality, laying hens, lysine, methionine, productive performance

## Abstract

**Background:**

Most laying hen diets are based on corn–soybean meal, in which methionine and lysine are limiting amino acids.

**Objectives:**

This study aimed to investigate on the effects of digestible lysine (dLys) and total sulphur amino acids (dTSAA) levels in the diet on productive performance of aged laying hens.

**Methods:**

A total of 384 Shaver White laying hens aged 105‒116 weeks were used in a randomized complete block design as a 2×4 factorial, with 8 treatments and 6 replicates of 8 hens each. Main effects included dLys concentrations of 0.75% and 0.80% and dTSAA of 0.70%, 0.73%, 0.76% and 0.79% of diet.

**Results:**

Diet containing 0.80% dLys and 0.73% dTSAA increased egg weight (EW) during 109‒112 week and overall period (*p* < 0.05). The interaction effects of dLys and dTSAA at the levels of 0.80 dLys along with 0.73% dTSAA resulted in the highest EW at the age of 105‒108 weeks (*p* < 0.05). Egg mass, egg production, feed intake, feed conversion ratio and egg quality indices including albumen quality, shell thickness, yolk, albumen and shell percentages were not significantly affected by the different levels of dLys and dTSAA in the diet.

**Conclusions:**

Increasing the level of dTSAA in the diet increased EW, but increasing the level of dLys had no significant effect on this index. The highest EW was observed in dLys 0.80% with dTSAA 0.73% and the lowest EW was observed in dLys 0.75% with dTSAA 0.79% during 105–116 weeks of age. The highest egg specific gravity was observed in the dLys level 0.75% with dTSAA 079%.

## Graphical abstract

Increasing the level of digestible sulphur‐containing amino acids in the diet of older laying hens increased egg weight, but increasing the level of digestible lysine did not have a significant effect on this index.

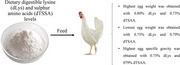

## 1 Introduction

Most laying‐hen diets are formulated based on corn and soybean meal, where methionine and lysine are the first and second limiting amino acids. Therefore, adding amino acids increases the efficiency of protein utilization in the diet (Macelline et al. 2021). The performance of laying hens is strongly influenced by the amount of methionine and lysine consumed daily. Several studies have been conducted to determine the methionine and lysine requirements in laying hens, and different values have been reported (Macelline et al. 2021).

The literature shows that the range of recommended values for methionine and lysine requirements is wide, and more research is needed in this area (Akbari Moghaddam Kakhki et al. 2023). Dietary protein efficiency is most effective when the levels of dietary amino acids are adjusted to the animal's needs (Macelline et al. 2021). Formulating dietary amino acids based on bird requirements reduces feed costs, meets the birds’ actual amino acid needs and minimizes nitrogen excretion and environmental pollution (Silva et al. 2015).

As commercial laying hens are increasingly retained for extended laying cycles, there is growing need to better understand how ageing affects their nutritional requirements, especially with respect to amino acids. Older hens (post‐peak) typically exhibit declines in feed efficiency, lower egg production rates and deteriorations in egg quality parameters such as shell strength and albumen consistency outcomes that are intimately linked to protein and amino acid supply. However, much of the established amino acid requirement data are derived from peak‐production hens, and these may not adequately reflect the altered metabolic demands of aged birds. Studies have highlighted inconsistencies in recommended levels of lysine, sulphur‐containing amino acids, threonine, tryptophan, branched‐chain amino acids and arginine among different ages, basal diets and strains, suggesting age effects are under‐accounted for in many models (Macelline et al. 2021). Further, research on old ISA Brown hens from 62 to 74 weeks shows that optimizing digestible lysine and sulphur amino acids improves eggshell quality and helps control egg weight during later lay (Akbari Moghaddam Kakhki et al. 2023). Thus, refining amino acid requirements for older hens is critical for sustaining productivity and egg quality in longer production cycles, aligning nutrition more closely with industry goals of cost efficiency, product stability and animal welfare.

Silva et al. (2015) reported that the requirement for lysine to achieve the best feed conversion ratio (FCR) is greater than that required to achieve maximum production. In addition, Akbari Moghaddam Kakhki et al. (2016b) reported that increasing lysine in the diet increased the percentage of egg production. Other studies conducted under specific conditions, such as the age of laying hens, the use of low‐quality protein and low ambient temperature, have shown a similar positive effect when increasing synthetic amino acids to levels above the NRC (1994) recommendation. However, there are reports on the performance of laying hens that do not fully support such results, including findings that supplementing with lysine levels higher than the NRC (1994) recommendation does not show any difference in production.

Regarding the effects of dietary lysine and methionine concentrations on EW, the reports are relatively more uniform, with most emphasizing the positive effects of these two amino acids at appropriate levels and ratios on EW (Akbari Moghaddam Kakhki et al. 2023).

The aim of this study was to investigate the effects of dietary digestible lysine (dLys) and total sulphur amino acids (dTSAA) concentrations on productive performance and egg quality traits in aged laying hens.

## 2 Materials and Methods

### 2.1 Birds, Diets and Management

A total of 384 Shaver White laying hens were utilized in this study. The experimental period included a two‐week pre‐experimental adaptation phase from weeks 103 to 105, followed by three 28‐day recording periods from weeks 105 to 116 of the laying cycle. The study employed a randomized complete block design with 8 treatments, 6 replications and 8 hens per replication. Each row of cages served as a block to minimize the impact of cage location. The basal diet was formulated according to the Shaver White Commercial Layer Management Guide (Hendrix Genetics 2024), ensuring all nutrients were provided except for lysine and sulphur‐containing amino acids, which were included at the lowest tested levels. Lysine and sulphur‐containing amino acid levels were adjusted by adding lysine and methionine to the basal diet (Table 1). l‐Lys supplementation levels were set at 0.75% and 0.80% of the basal diet, while DL‐Met supplementation levels included 0.70%, 0.73%, 0.76% and 0.79%.

**TABLE 1:** Ingredients and nutrient composition of basal diet during 105–117 weeks; as fed basis.

Ingredients	(%)
Corn	66.18
Soybean meal (42% CP)	19.66
Calcium carbonate	10.91
Soy oil	0.75
Dicalcium phosphate	1.33
Common salt	0.28
Sodium bicarbonate	0.10
DL‐ Methionine	0.19
l‐Lysine HCl	0.10
Vitamin premix^a^	0.25
Mineral premix^b^	0.25
Calculated values, %	
Metabolizable energy (kcal/kg)	2700
Crude protein	14.5
Calcium	4.5
Available phosphorous	0.38
Sodium	0.18
Digestible methionine	0.42
Digestible Met + Cys	0.60
Digestible lysine	0.72
Digestible threonine	0.56

^a^Provided per kg of diet: vitamin A (retinol), 8,800 IU; vitamin D3 (cholecalciferol), 3,300 IU; vitamin E (DL‐α‐tocopheryl acetate), 18.5 IU; vitamin K3 (menadione), 2.2 mg; vitamin B1 (thiamin), 2.2 mg; vitamin B2 (riboflavin), 5.5 mg; vitamin B3 (niacin), 28.0 mg; vitamin B5 (pantothenic acid), 6.6 mg; vitamin B6 (pyridoxine), 3.5 mg; vitamin B9 (folic acid), 0.7 mg; vitamin B12 (cyanocobalamin), 0.02 mg; biotin, 0.05 mg; antioxidant 1.0 mg.

^b^ Provided mg/kg of diet: Mn (manganese sulphate) 80.0, Fe (iron sulphate) 75.0, Zn (zinc sulphate) 64.0, Cu (copper sulphate) 6.0, Se (sodium selenite) 0.3.

The chemical composition of the diet ingredients and the complete diet was determined based on AOAC (2005) methods. Samples were ground and analysed for crude protein (Kjeldahl; N × 6.25; method 990.03), dry matter (DM; method 930.15) and total ash (method 942.05). Calcium (Ca) and total phosphorus (P) levels in the diet were measured using Inductively Coupled Plasma‐Optical Emission Spectroscopy (ICP‐OES) (Spectro Arcos, Kleve, Germany; method 968.08).

Cage dimensions were 60 cm in length, 60 cm in width and 40 cm in height, with a slope of 7.8°. Each cage was equipped with two nipple drinkers and one trough feeder at the front. Throughout the experiment, birds had ad libitum access to feed and water. The lighting schedule consisted of 16 h of light (intensity of 3.5 watts/m^2^ of floor area) and 8 h of darkness. The room temperature was maintained between 16°C and 18°C. Each cage housing 8 birds was considered an experimental unit, and wood partitions were used to prevent cross‐feeding between replicate cages.

### 2.2 Productive Performance

Eggs were collected daily at 7:00 AM and weighed using a digital scale (0.001‐g precision, model GF 400, A&D Weighing, San Jose, CA, USA). Egg production and egg mass were calculated daily using the following formulas:

Egg production (%) = (number of eggs in replicate / number of hens in replicate) × 100

Egg mass (g/hen/d) = (egg weight in replicate × egg production % in replicate) / 100

Feed intake (FI) was calculated weekly, and the FI for each experimental unit over the entire experimental period was averaged over 12 weeks. At the end of each week, feed added to each experimental unit was weighed and recorded before being gradually poured into the feeder. The amount of feed consumed was determined by the difference between the weight of feed added and the remaining feed in the feeder. All calculations accounted for the weight of the bag used for measuring.

At the end of each week, all laid eggs were weighed for each replicate cage using a digital balance (0.001‐g precision, model GF 400, A&D Weighing, San Jose, CA, USA). The mean EW for each experimental unit over the entire experimental period was calculated from the average of 12 weeks. The FCR was determined by dividing the feed consumed (g) by the weight of eggs produced (g) for each experimental unit at the end of each week. The overall FCR for each experimental unit during the entire experimental period was calculated by dividing the total feed consumed (g) by the total weight of eggs produced (g) during the same period (Alsherify and Hassanabadi 2024). No mortality occurred during the experiment.

### 2.3 Egg Quality Traits

To assess quality traits, 3 eggs were randomly selected from each experimental unit at the end of each 28‐day period. Egg weight was recorded using a digital balance (0.001‐g precision, model GF 400, A&D Weighing, San Jose, CA, USA). Specific gravity was measured by immersing the egg in water using a small mesh made from copper wire attached to a hook on the scale. The egg was fully submerged, and its weight in distilled water was recorded. Room and water temperatures were kept constant throughout the measurement, and water was replaced regularly. The formula for calculating specific gravity is:

Specific gravity = (Egg weight in air) / (Egg weight in air − Egg weight when submerged in water)

The average specific gravity of eggs from each experimental unit over the entire experimental period was computed from the three 28‐day periods.

To measure egg shape index, three eggs from each experimental unit at the end of each 28‐day period were measured using a caliper with an accuracy of 0.01 mm (0.01‐mm digital caliper, model 1116–150, Insize Co, Suzhou, China). The shape index was calculated using the formula:

Shape index = Egg width (mm) / Egg height (mm)

The egg shape index for each experimental unit throughout the entire experimental period was derived from the average of the three 28‐day periods.

To determine the percentage of internal components of eggs (yolk, albumen and shell), three eggs were randomly selected from each experimental unit at the end of each 28‐day period, and the weights of the yolk, albumen and shell were measured. The method described by Prochaska et al. (1996) was used, where yolk and albumen were separated, and excess albumen was removed from the yolk's surface with a paper towel. The weight of the albumen was calculated as the difference between the total EW and the sum of the yolk and shell weights. The shell weight was measured after washing with distilled water and air exposure for 48 h. Weighing was performed using a digital balance (0.001‐g precision, model GF 400, A&D Weighing, San Jose, CA, USA). The weights of the yolk, albumen and shell were expressed as percentages of the total EW, and the averages for each experimental unit across the entire experimental period were derived from the three 28‐day periods.

The height of the egg white was measured with a height gauge accurate to 0.01 mm (0.01‐mm digital caliper, Insize Co, Suzhou, China), and the Haugh unit (HU) was calculated using the following formula (Haugh, 1937):

HU = 100 × log10(*H* + 7.57 − 1.7 *W*^0.37)

 where *H* is the height of the albumen in mm and *W* is the weight of the egg in grams. The HU for each experimental unit over the entire experimental period was calculated from the average of the three 28‐day periods.

Eggshell thickness was measured with a digital micrometre (0.01‐mm digital micrometre, model 293‐240, Mitutoyo Co, Kanagawa, Japan) at three points: Middle, top and bottom of the shell (Carter 1975). The average of these measurements was considered the eggshell thickness, calculated for each experimental unit over the entire experimental period based on the average of the three 28‐day periods.

### 2.4 Statistical Analysis

Data were tested for normality using SAS (version 9.4, SAS Institute, 2012) via the Univariate plot normal procedure. Non‐normal data were transformed to arcsine for normalization. Subsequently, data were analysed using the General Linear Model (GLM) procedure of SAS in a randomized complete block design as a 2 × 4 factorial arrangement (2 levels of dLys at 0.75% and 0.80% of the diet, and 4 levels of dTSAA at 0.70%, 0.73%, 0.76% and 0.79% of the diet). Means were compared using Tukey's test at a significance level of 0.05.

## 3 Results

The effects of different dietary concentrations of dLys and dTSAA, as well as their interaction, on EW in laying hens are summarized in Table 2. Neither the block effect nor the dLys level in the diet significantly impacted EW during any of the experimental periods or over the entire study. However, the main effect of dietary dTSAA concentration significantly influenced EW during the 105–108 and 109–112 week periods, with the highest EW observed at a dTSAA level of 0.79% of the diet. Significant interactions between dLys and dTSAA levels on EW were noted in both the first and second (*p* < 0.05) experimental periods, with the highest EW recorded in layers fed diets containing 0.80% l‐Lys and 0.73% dTSAA. Conversely, the lowest EW was observed at a dLys level of 0.75% and dTSAA level of 0.79% during the 105–108 and 109–112 week periods.

**TABLE 2:** The effect of different dietary levels of digestible lysine (dLys) and sulphur amino acids (dTSAA) on the egg weight (g) in Shaver White laying hens at different ages.

Age (week)					Treatment
Overall	113–116	109–112	105–108	% of diet	Main effects
(105–116)					
62.7	62.4	63.3	62.4	0.75	dLys
62.2	62.3	62.6	61.7	0.8	
0.66	0.3	0.39	0.62		SEM
61.9	62.8	63.2^ab^	59.8^c^	0.7	dTSAA
61.9	62.4	62.5^ab^	60.7^bc^	0.73	
62.5	62.1	62.0^b^	63.4^ab^	0.76	
63.5	62.2	64.2^a^	64.2^a^	0.79	
0.67	0.3	0.39	0.62		SEM
					Interaction
				dTSAA	dLys ×
62.8	62.9	63.5^ab^	62.0^ab^	0.7	0.75
63.5	62.7	63.1^ab^	64.6^a^	0.73	
62.9	63	63.3^ab^	62.3^ab^	0.76	
59.8	61.2	60.8^b^	57.5^c^	0.79	
62.3	61.5	62.5^b^	63.0^ab^	0.7	0.8
64.8	63	65.8^a^	65.5^a^	0.73	
62.8	63	63.1^ab^	62.2^ab^	0.76	
60.9	61.8	61.5^b^	59.3^bc^	0.79	
0.88	0.69	0.38	0.59		SEM
*p* value					
0.3	0.58	0.75	0.87		Block
0.26	0.84	0.3	0.51		dLys
0.23	0.76	0.02	0.01		dTSAA
0.09	0.12	0.02	0.04		dLys × dTSAA

^a, b^ Means without common superscript within a column are significantly different (*p <* 0.05).

The effects of dLys and dTSAA concentrations on average egg mass (g/hen/d) in Shaver White laying hens at different ages are summarized in Table 3. The block effect was significant (*p* < 0.05); however, the treatments did not significantly affect egg mass (*p* > 0.05).

**TABLE 3:** The effect of different dietary levels of digestible lysine (dLys) and sulphur amino acids (dTSAA) on the egg mass (g/hen/d) in Shaver White laying hens at different ages.

Age (week)			Treatment			
Overall						
(105–116)	113–116	109–112	105–108		% of diet	Main effects
43.3	43.7	43.7	42.6		0.75	dLys
43.1	44.4	43.4	41.6		0.80	
1.17	1.23	1.11	1.01			SEM
41.9	42.2	42.3	41.1		0.70	dTSAA
44.4	45.7	45.0	42.5		0.73	
42.6	43.7	42.9	41.1		0.76	
44.1	44.6	44.1	43.7		0.79	
1.17	1.33	1.10	1.01			SEM
						Interaction
					dTSAA	dLys ×
46.2	47.3	46.7	44.7		0.70	0.75
44.5	44.2	44.9	44.4		0.73	
39.3	40.3	39.8	37.9		0.76	
39.6	39.9	40.2	38.6		0.79	
45.2	45.7	45.1	44.7		0.70	0.80
43.1	43.5	43.1	42.8		0.73	
45.5	47.5	45.5	43.6		0.76	
42.5	44.0	43.3	40.3		0.79	
2.40	2.50	2.29	2.04			SEM
*p* value						
0.007	0.01	0.006	0.21			Block
0.67	0.77	0.87	0.58			dLys
0.77	0.75	0.76	0.66			dTSAA
0.37	0.27	0.21	0.08			dLys × dTSAA

The effects of varying dietary levels of dLys and dTSAA, along with their interaction, on egg production percentage in laying hens are presented in Table 4. Neither dietary concentrations of dLys nor dTSAA, nor their interaction, significantly influenced egg production (*p* > 0.05).

**TABLE 4:** The effect of different dietary levels digestible lysine (dLys) and sulphur amino acids (dTSAA) on the hen day egg production (%) in Shaver White laying hens at different ages.

Age (week)					Treatment
Overall (105–116)	113–116	109–112	105–108	% of diet	Main effects
69.1	69.9	69.1	68.4	0.75	dLys
69.4	71.2	69.4	67.5	0.8	
1.91	2.05	1.23	1.5		SEM
66.3	67.2	67	64.8	0.7	dTSAA
71.6	73.1	71.9	69.9	0.73	
69.2	70.1	69	68.6	0.76	
69.7	71.7	69	68.4	0.79	
1.89	2.04	1.73	2.49		SEM
					Interaction
				dTSAA	dLys ×
73.5	75.1	73.5	72	0.7	0.75
70.1	70.4	71	68.8	0.73	
62.7	64.1	63	60.9	0.76	
66	64.9	66	67.1	0.79	
72.4	74.3	72.1	70.8	0.7	0.8
67	69.1	66	65.8	0.73	
72.5	75.4	72.1	70.1	0.76	
69.8	71.2	70.3	67.9	0.79	
3.25	3.32	2.02	2.57		SEM
*p* value					
0.003	0.01	0.007	0.12		Block
0.94	0.7	0.93	0.71		dLys
0.72	0.67	0.71	0.54		dTSAA
0.27	0.31	0.25	0.26		dLys × dTSAA

Table 5 summarizes the effects of different dietary levels of dLys and dTSAA, and their interaction, on FI in laying hens. No significant influence on FI was observed during any of the experimental periods or over the entire study (*p* > 0.05).

**TABLE 5:** The effect of different dietary levels of digestible lysine (dLys) and sulphur amino acids (dTSAA) on the feed intake (g/hen/d) in Shaver White laying hens at different ages.

Age (week)					Treatment
**Overall**	**113–116**	**109–112**	**105–108**	**% of diet**	**Main effects**
**(105–116)**					
117.6	121.7	117.2	114	0.75	dLys
117.3	121.6	116.8	113.4	0.8	
0.58	0.69	0.44	0.75		SEM
117.4	122.1	117.2	113	0.7	dTSAA
117.7	122.1	117.6	113.4	0.73	
116.4	120.9	116.2	112.2	0.76	
118.3	121.5	117	116.3	0.79	
1.57	1.69	1.44	1.77		SEM
					Interaction
				dTSAA	dLys ×
118.1	122.7	118.1	113.6	0.7	0.75
118.5	122.5	117.9	115.2	0.73	
116.4	121.8	116.5	110.8	0.76	
115.5	120.7	116	109.9	0.79	
118.1	122	117.2	115.2	0.7	0.8
118.4	121	116.9	117.3	0.73	
117.3	121	116.4	114.5	0.76	
117.3	121.6	117.2	113.2	0.79	
1.58	1.79	1.46	1.92		SEM
*p* value					
0.34	0.01	0.14	0.003		Block
0.55	0.94	0.61	0.66		dLys
0.39	0.85	0.59	0.16		dTSAA
0.84	0.92	0.77	0.13		dLys × dTSAA

The effect of varying levels of dLys and dTSAA in the diet on FCR in Shaver White laying hens is summarized in Table 6. Treatments did not significantly affect FCR during any of the experimental periods or the overall study duration (*p* > 0.05).

**TABLE 6:** The effect of different dietary levels of digestible lysine (dLys) and sulphur amino acids (dTSAA) on the feed conversion ratio (g/g) in Shaver White laying hens at different ages.

		Treatment			Age (week)
Main effects	% of diet	105–108	109–112	113–116	Overall (105–116)
dLys	0.75	2.68	2.68	2.68	2.71
	0.80	2.73	2.69	2.73	2.72
SEM		0.07	0.09	0.10	0.13
dTSAA	0.70	2.75	2.77	2.75	2.80
	0.73	2.67	2.61	2.67	2.65
	0.76	2.73	2.71	2.73	2.74
	0.79	2.66	2.65	2.66	2.68
SEM		0.08	0.10	0.11	0.13
Interaction					
dLys ×	dTSAA				
0.75	0.70	2.54	2.53	2.54	2.56
	0.73	2.59	2.63	2.59	2.66
	0.76	2.92	2.93	2.92	2.96
	0.79	2.85	2.89	2.85	2.92
0.80	0.70	2.58	2.60	2.58	2.62
	0.73	2.74	2.71	2.74	2.74
	0.76	2.63	2.56	2.63	2.58
	0.79	2.81	2.71	2.81	2.76
SEM		0.80	0.41	0.53	0.25
					*p* value
Block		0.25	0.01	0.003	0.03
dLys		0.92	0.61	0.47	0.59
dTSAA		0.95	0.85	079	0.76
dLys × dTSAA		0.16	0.28	0.32	0.42

Table 7 presents the effects of different dietary levels of dLys and dTSAA, and their interaction, on egg quality traits in Shaver White laying hens. Dietary levels of dLys and dTSAA, along with their interaction, did not significantly influence yolk, white or eggshell percentages, HU or eggshell thickness (*p* > 0.05). However, the interaction effect of dietary levels of dLys and dTSAA on egg specific gravity was statistically significant (*p* < 0.05); increasing dietary dTSAA levels at a 0.75% dLys level enhanced this index, while at a 0.80% dLys level, this response was not observed. The main effects of dLys and dTSAA did not significantly affect egg specific gravity (*p* > 0.05).

**TABLE 7:** The effect of different dietary levels of digestible lysine (dLys) and sulphur amino acids (dTSAA) on the egg quality traits in Shaver White laying hens during 105–116 weeks of age.

					Treatment		
Main effects	% of diet	Specific gravity (g/cm^3^)	Yolk (%)	Shell (%)	Shell thickness (mm)	Haugh unit	Albumen index (%)
							
dLys	0.75	1.09	27.2	11.6	0.39	82.3	82.5
	0.80	1.09	27.6	11.5	0.39	81.5	82.0
SEM		0.01	0.15	0.08	0.01	0.44	0.44
dTSAA	0.70	1.09	27.7	11.5	0.39	83.0	82.6
	0.73	1.09	27.3	11.5	0.38	80.6	82.4
	0.76	1.09	27.4	11.7	0.39	80.0	82.2
	0.79	1.09	27.3	11.5	0.39	82.0	81.8
SEM		0.01	0.15	0.08	0.01	1.48	0.46
Interaction							
dLys ×	dTSAA						
0.75	0.70	1.087^b^	26.8	11.4	0.38	81.2	83.2
	0.73	1.090^ab^	27.5	11.5	0.39	83.1	82.3
	0.76	1.088^ab^	27.9	11.4	0.39	82.7	82.9
	0.79	1.092^a^	27.5	12.0	0.39	83.6	82.9
0.80	0.70	1.088^ab^	27.4	11.5	0.40	82.7	82.1
	0.73	1.087^b^	27.1	11.5	0.39	81.2	81.4
	0.76	1.087^b^	27.4	11.4	0.39	80.5	81.5
	0.79	1.088^ab^	27.7	11.7	0.39	80.0	81.5
SEM		0.001	0.14	0.19	0.02	1.11	1.09
							*p* value
Block		0.005	0.95	0.27	0.49	0.00	0.13
dLys		0.12	0.16	0.58	0.89	0.31	0.55
dTSAA		0.47	0.59	0.79	0.39	0.26	0.89
dLys × dTSAA		0.05	0.54	0.22	0.36	0.27	0.59

^a, b^ Means without common superscript within a column are significantly different (*p <* 0.05).

## 4 Discussion

Randomized complete block design used in this study aimed to minimize location‐related variability, the significance of block effects for some variables (egg mass, egg production and FI) suggests that micro‐environmental differences among cage rows such as light intensity, air movement or feed distribution may have contributed to residual variation. These findings emphasize the importance of accounting for potential spatial heterogeneity in experimental housing environments. Future studies could apply mixed‐model approaches, where cage or row effects are treated as random factors, to better partition environmental variance and improve statistical robustness (Macelline et al. 2021).

Production performance traits are critical in the laying hen industry. In this study, FI was unaffected by the treatments. Notably, variations in FI between treatments can confound results. Pérez‐Bonilla et al. (2012) reported that reducing energy intake from 324 to 304 kcal/bird/d led to a linear decrease in egg production and EW in 59‐week‐old Hy‐Line brown laying hens. Dietary amino acid imbalances can lead to changes in blood amino acid levels that negatively affect appetite (Wang et al. 2020). Thus, it appears that the addition of different levels of lysine and methionine in this experiment did not result in amino acid imbalances.

High daily egg production and FCR were not significantly affected by the main effects of dietary dLys and dTSAA concentrations or their interaction. Reported estimates of dTSAA requirements for optimal egg production performance range from 565 to 621 mg/bird/d (Macelline et al. 2021; Macelline et al. 2022). Variations in amino acid requirements can be attributed to differing experimental conditions, including breed, age, housing system, basal diet, statistical models and selected production criteria (Akbari Moghaddam Kakhki et al. 2023). In markets where egg producers are compensated based on egg count, maintaining egg count while managing egg size is an ideal strategy for eggshell quality. The lack of effect of varying levels of dLys and dTSAA on egg production aligns with this strategy.

In this study, no significant differences in FCR were observed among the experimental groups, which contrasts with findings by Akbari Moghaddam Kakhki et al. (2016a, 2016b) and Spangler et al. (2019), likely due to differences in the age of the hens. Some researchers have shown that increased dietary methionine intake significantly improves FCR (Bunchasak and Silapasorn 2005), which contradicts our findings.

While the main effect of dietary dLys concentration did not significantly affect EW, increasing dietary dTSAA concentration positively impacted this parameter, with their interaction being significant (*p* < 0.05). This finding underscores the importance of considering amino acid interactions when designing nutritional strategies for managing EW. However, as the number of marketable eggs ultimately dictates profitability, maintaining high egg production levels is crucial. Domingues et al. (2012) reported that EW was significantly influenced by lysine and methionine supplementation during the second phase of laying, noting that hens fed diets with 0.56% digestible lysine and 0.56% digestible methionine + cysteine exhibited higher egg weights due to adequate amino acid supply. Our study aligns with Harms and Russell (2003), who observed increased EW with higher levels of methionine + cysteine in the diet. Conversely, our results regarding dietary dLys concentration diverge from those of Akbari Moghaddam Kakhki et al. (2016b), who reported that increasing dietary lysine from 0.657% to 0.757% increased average EW. This discrepancy may be due to the difference in the magnitude of dietary lysine increase in their study compared to ours.

The lack of significant effect of dietary lysine on egg weight in the present study, despite previous reports showing positive responses (Akbari Moghaddam Kakhki et al. 2016b; Harms and Russell 2003), may be attributed to multiple interacting factors beyond hen age alone. First, aged hens often exhibit reduced intestinal absorption efficiency and altered amino acid metabolism, which can decrease the utilization of supplemental lysine for protein synthesis and albumen deposition (Park and Sohn 2018). Moreover, long‐term adaptation to consistent amino acid supply across prolonged laying cycles may induce metabolic adjustments that stabilize egg size despite incremental lysine increases. The protein quality and amino acid balance of the basal corn–soybean meal diet could also have influenced responsiveness; when other essential amino acids are not limiting, additional lysine supplementation may not further enhance egg weight due to balanced protein accretion efficiency (Macelline et al. 2021). Breed‐specific differences may further contribute Shaver White hens, for instance, tend to maintain egg production rate and persistency rather than egg size, differing from other strains such as ISA Brown (Akbari Moghaddam Kakhki et al. 2023). Collectively, these factors may suggest that in older layers, the marginal efficiency of dietary lysine for egg mass synthesis declines due to physiological adaptation and reduced anabolic capacity, rather than an absolute deficiency of this amino acid.

In our study, different dietary concentrations of dLys and dTSAA did not significantly affect egg mass produced. Similarly, Tepox Pérez et al. (2012) found no significant effects of added sulphur‐containing amino acids on egg mass in commercial laying hens. Variations in amino acid requirements can result from differing experimental conditions, including breed, age, housing system, basal diet, statistical model and selected production criteria (Akbari Moghaddam Kakhki et al. 2023).

In this study, dietary treatments did not affect egg production percentage in hens aged 105–116 weeks. Tepox Pérez et al. (2012) also found no differences in egg production percentage at higher levels of methionine + cysteine fed to commercial hens near peak production. Akbari Moghaddam Kakhki et al. (2016b) reported that varying dietary concentrations of lysine improved egg production percentage, which is inconsistent with our findings. The differing results may relate to age differences in the hens and lower amino acid requirements at older ages; our study involved hens aged 105–116 weeks, while their study examined hens aged 32–44 weeks.

Results from this experiment indicated that eggshell thickness in hens aged 105–116 weeks was not significantly affected by treatments (*p* < 0.05). Eggshell thickness is a critical quality trait in commercial laying hens, as thin‐shelled eggs can break during transportation. Several factors influence shell thickness, including the role of sulphur‐containing amino acids in eggshell quality. Increased sulphate groups in the shell protein matrix enhance calcium‐binding ability, improving both shell strength and overall quality (Novak et al. 2004). Variations in hens’ responses to dietary dLys and dTSAA levels regarding eggshell thickness reported in the literature appear to correlate with hen age. Dietary dTSAA levels had a linear effect on eggshell thickness in Lohmann hens at 34 weeks of age, while a quadratic effect was noted on eggshell percentage and thickness in 50‐week‐old hens (Carvalho et al. 2018). To address poor eggshell quality in older hens, understanding the underlying causes is crucial. Ageing leads to uterine tissue damage and decreased secretion of gonadal hormones, resulting in lower uterine calcium levels, which can impair basement membrane crystallization and mammary gland formation (Park and Sohn 2018). These changes lead to reduced mammary gland density and slower reticulum formation. In addition, elevated sodium levels later in the laying period contribute to poor eggshell formation in older birds (Park and Sohn 2018). Furthermore, as hens age, increasing egg size exacerbates eggshell issues. Rowland (1980) conducted one of the first studies on nutritional strategies to maintain eggshell quality, demonstrating that reducing dietary crude protein from 16% to 13.5% decreased the natural rate of egg weight gain and helped preserve eggshell quality. This study highlighted that dietary interventions could effectively maintain eggshell quality in laying hens and suggested that controlling the rate of egg weight gain without compromising egg production should be considered in developing strategies for eggshell quality maintenance (Roland 1980).

The HU is a significant index for evaluating internal egg characteristics. In this study, dietary dLys and dTSAA concentrations did not significantly affect the HU in 105 to 116‐week‐old Shaver White laying hens. Domingues et al. (2012) also reported no effect of varying lysine to methionine + cysteine ratios on egg HU in older laying hens. In contrast, Carvalho et al. (2018) found that HU was only affected in eggs from laying hens at 34 weeks of age, with optimal levels of digestible methionine + cysteine at 0.546% of the diet. It appears that hen age is a crucial factor in response to dLys and dTSAA levels in the diet.

Egg specific gravity is a trait influenced more by eggshell quality than by the internal contents of the egg. Typically, egg specific gravity ranges from 1.06 to 1.099; higher specific gravity indicates better shell quality. In this study, the interaction effect of dietary dLys and dTSAA concentrations on egg specific gravity was significant, with the highest specific gravity observed at 0.75% dLys and 0.79% dTSAA levels. This suggests that increasing dietary levels of sulphur‐containing amino acids in older hens may help maintain shell strength despite larger egg sizes, thereby preventing breakage and cracking. Petersen et al. (1983) similarly found that increasing methionine intake from 255 to 300 mg/hen/d improved eggshell quality, as evidenced by increased specific gravity in 50‐week‐old laying hens.

In this study, the relative weights of shell, yolk and albumen were not affected by varying levels of dLys and dTSAA in the diets of older laying hens. Hens require approximately 2.6 mg of digestible methionine to produce 1 g of egg (Gomez and Angeles 2009). Changes in methionine intake can affect egg, albumen and yolk weights (Bunchasak and Silapasorn 2005). Similarly, Carvalho et al. (2018) reported no effect of dietary sulphur amino acid levels on yolk and albumen percentages in eggs from laying hens.

The current goal in laying hen production is to raise long‐lived hens capable of producing 500 eggs in 100 weeks without molting (Bain et al. 2016). Reduced eggshell quality can hinder the production of 500 marketable eggs. Adjusting dietary amino acid levels may lead to deviations from optimal EW, but this could be more profitable due to improved eggshell quality and reduced instances of degraded eggs. In this study, increasing dietary methionine levels enhanced EW, and the significant interaction effect of dietary dLys and dTSAA levels on EW indicates that optimal levels of these two amino acids should be considered concurrently. Careful examination of potential effects on various performance metrics is essential when implementing dietary amino acid strategies aimed at improving eggshell quality in commercial laying hens.

Although most egg quality traits such as yolk, albumen and shell percentages, HU and shell thickness were not significantly influenced by dietary dLys and dTSAA levels, this stability suggests that amino acid variations within the tested range were sufficient to maintain normal physiological processes related to egg formation in aged hens. The lack of response in these parameters may reflect a plateau effect, where the amino acid supply already met or slightly exceeded the hens’ maintenance and production needs, leading to no further improvement in quality indices. In contrast, the significant interaction between dLys and dTSAA levels for egg specific gravity indicates that sulphur amino acids may still play a crucial role in maintaining eggshell integrity at later stages of production. Higher specific gravity observed at 0.75% dLys with 0.79% dTSAA suggests enhanced shell density and reduced porosity, which could improve egg resistance to cracking during collection, transport and storage factors of particular importance for the marketability of eggs from older flocks. This interaction likely reflects the role of dTSAA in the synthesis of matrix proteins essential for calcium binding during shell formation. Therefore, maintaining an adequate balance of dLys and dTSAA may help mitigate age‐related declines in shell quality and preserve the handling properties of eggs in commercial production.

## 5 Conclusions

Different concentrations of dLys and dTSAA in the diet had no significant effects on productive performance metrics such as egg mass, egg production percentage, FI and FCR, except for egg weight. In addition, egg quality indices were not significantly affected by the treatments, except for specific gravity. The findings of this study suggest that regulating dLys and dTSAA may be a viable strategy for managing egg weight in aged laying hens without compromising egg production. It is recommended that breeders of the aged Shaver White strain use diets containing 0.80% dLys and 0.73% dTSAA when aiming to produce larger eggs. Conversely, to achieve smaller egg sizes, hens should be fed diets containing 0.75% lysine and 0.79% dTSAA. However, these findings may not be directly applicable to other strains, all production ages, or all rearing systems and should be interpreted with caution.

## Author Contributions

**Ahmad Hassanabadi**: project administration, supervision, software, methodology, conceptualization, funding acquisition, writing – review and editing. **Elnaz Fallah Moghadam**: investigation, data curation. **Heydar Zarghi**: advising, review and editing.

## Funding

This research was supported by the Ferdowsi University of Mashhad [project number 3/60610].

## Ethics Statement

The authors confirm that the ethical policies of the journal, as noted in the journal's author guidelines page, have been adhered to and the appropriate ethical review committee approval has been received. The authors confirm that they have followed EU standards for the protection of animals used for scientific purposes and feed legislation. Also, this study was conducted according to the procedures established by the Iranian Ministry of Agriculture (Experimental Authorization No. ASRI‐2016‐95014).

## Conflicts of Interest

The authors declare no conflicts of interest.

## Data Availability

The data that support the findings of this study are available from the corresponding author upon reasonable request.

## References

[vms370789-bib-0001] Akbari Moghaddam Kakhki, R. , C. Alfonso‐Carrillo , and A. I. García‐Ruiz . 2023. “The Impact of Digestible Lysine and Sulfur Amino Acids on Eggshell Quality and Egg Weight Control in Old ISA Brown Hens During 62 to 74 Wk.” Poultry Science 102, no. 9: 102860. 10.1016/j.psj.2023.102860 PMC10466235 37406436

[vms370789-bib-0002] Akbari Moghaddam Kakhki, R. , A. Golian , and H. Zarghi . 2016a. “Effect of Digestible Methionine+ Cystine Concentration on Performance, Egg Quality and Blood Metabolites in Laying Hens.” British Poultry Science 57, no. 3: 403–414. 10.1080/00071668.2016.1173199 27074313

[vms370789-bib-0003] Akbari Moghaddam Kakhki, R. , A. Golian , and H. Zarghi . 2016b. “Effect of Dietary Digestible Lysine Concentration on Performance, Egg Quality, and Blood Metabolites in Laying Hens.” Journal of Applied Poultry Research 25, no. 4: 506–517. 10.1080/00071668.2016.1173199 27074313

[vms370789-bib-0004] Alsherify, S. M. , and A. Hassanabadi . 2024. “Effects of Adding Different Levels of Fructooligosaccharide to Diet on Productive Performance, Egg Quality Traits, Immune Response and Blood Metabolites in Commercial Laying Hens.” Veterinary Medicine and Science 10, no. 5: e1550. 39119788 10.1002/vms3.1550 PMC11310662

[vms370789-bib-0005] AOAC . 2005. Official Methods of Analysis. 18th ed. AOAC International.

[vms370789-bib-0006] Bain, M. M. , Y. Nys , and I. C. Dunn . 2016. “Increasing Persistency in Lay and Stabilising Egg Quality in Longer Laying Cycles. What Are the Challenges?.” British Poultry Science 57, no. 3: 330–338. 10.1080/00071668.2016.1161727 PMC4940894 26982003

[vms370789-bib-0007] Bunchasak, C. , and T. Silapasorn . 2005. “Effects of Adding Methionine in Low‐Protein Diet on Production Performance, Reproductive Organs and Chemical Liver Composition of Laying Hens Under Tropical Conditions.” International Journal of Poultry Science 4, no. 5: 301–308.

[vms370789-bib-0008] Carter, T. C. 1975. “The Hen's Egg: Estimation of Shell Superficial Area and Egg Volume, Using Measurements of Fresh Egg Weight and Shell Length and Breadth Alone or in Combination.” British Poultry Science 16: 541–543.

[vms370789-bib-0009] Carvalho, T. S. M. , L. S. Sousa , F. A. Nogueira , et al. 2018. “Digestible Methionine+ Cysteine in the Diet of Commercial Layers and Its Influence on the Performance, Quality, and Amino Acid Profile of Eggs and Economic Evaluation.” Poultry Science 97, no. 6: 2044–2052. 10.3382/ps/pey036 29546372

[vms370789-bib-0010] Domingues, C. D. F. , S. Sgavioli , M. F. F. M. Praes , et al. 2012. “Lysine and Methionine+ Cystine for Laying Hens During the Post‐Molting Phase.” Brazilian Journal of Poultry Science 14: 187–192.

[vms370789-bib-0011] Hendrix Genetics . 2024. “Shaver White Commercial Layer Management Guide.” https://layinghens.hendrix‐genetics.com/en/.

[vms370789-bib-0012] Gomez, S. , and M. Angeles . 2009. “Effect of Threonine and Methionine Levels in the Diet of Laying Hens in the Second Cycle of Production.” Journal of Applied Poultry Research 18, no. 3: 452–457.

[vms370789-bib-0013] Harms, R. H. , and G. B. Russell . 2003. “Performance of Commercial Laying Hens Fed Diets With Various Levels of Methionine.” Journal of Applied Poultry Research 12, no. 4: 449–455.

[vms370789-bib-0015] Haugh, R. R. 1937. “The Haugh Unit for Measuring Egg Quality.” United States Egg Poultry Magazine 43: 552–573.

[vms370789-bib-0016] Macelline, S. P. , M. Toghyani , P. V. Chrystal , et al. 2022. “Essential Amino Acid Recommendations for Isa Brown Layers During Peak and Post Peak Production.” Poultry Science 101, no. 12: 102171. 10.1016/j.psj.2022.102171 PMC9573913 36240635

[vms370789-bib-0017] Macelline, S. P. , M. Toghyani , P. V. Chrystal , P. H. Selle , and S. Y. Liu . 2021. “Amino Acid Requirements for Laying Hens: a Comprehensive Review.” Poultry Science 100, no. 5: 101036. 10.1016/j.psj.2021.101036 PMC8024705 33770542

[vms370789-bib-0018] National Research Council (NRC) . 1994. Nutrient Requirements of Poultry. 9th ed. National Academy Press.

[vms370789-bib-0019] Novak, C. , H. Yakout , and S. Scheideler . 2004. “The Combined Effects of Dietary Lysine and Total Sulfur Amino Acid Level on Egg Production Parameters and Egg Components in Dekalb Delta Laying Hens.” Poultry Science 83, no. 6: 977–984. 10.1093/ps/83.6.977 15206625

[vms370789-bib-0020] Park, J. A. , and S. H. Sohn . 2018. “The Influence of Hen Aging on Eggshell Ultrastructure and Shell Mineral Components.” Korean Journal for Food Science of Animal Resources 38, no. 5: 1080. 30479513 10.5851/kosfa.2018.e41 PMC6238045

[vms370789-bib-0021] Pérez‐Bonilla, A. , S. Novoa , J. García , M. Mohiti‐Asli , M. Frikha , and G. G. Mateos . 2012. “Effects of Energy Concentration of the Diet on Productive Performance and Egg Quality of Brown Egg‐Laying Hens Differing in Initial Body Weight.” Poultry Science 91, no. 12: 3156–3166. 10.3382/ps.2012-02526 23155026

[vms370789-bib-0022] Petersen, C. F. , E. A. Sauter , E. E. Steele , and J. F. Parkinson . 1983. “Use of Methionine Intake Restriction to Improve Egg Shell Quality by Control of Egg Weight.” Poultry Science 62, no. 10: 2044–2047.

[vms370789-bib-0023] Prochaska, J. F. , J. B. Carey , and D. J. Shafer . 1996. “The Effect of L‐lysine Intake on Egg Component Yield and Composition in Laying Hens.” Poultry Science 75, no. 10: 1268–1277. 10.3382/ps.0751268 8893305

[vms370789-bib-0024] Statistical Analyses System (SAS) . 2012. SAS/STAT Software, Version 9.4. Cary, NC: SAS Institute Inc.

[vms370789-bib-0025] Silva, E. P. , E. B. Malheiros , N. K. Sakomura , et al. 2015. “Lysine Requirements of Laying Hens.” Livestock Science 173: 69–77.

[vms370789-bib-0026] Spangler, H. , P. Utterback , C. M. Parsons , and P. Tillman . 2019. “Determining the Digestible Lysine Requirement of 22‐to 47‐Week‐Old Lohmann Laying Hens Using an Increasing Protein Titration Methodology.” Poultry Science 98, no. 4: 1706–1715. 10.3382/ps/pey503 30508158

[vms370789-bib-0027] Roland Sr, D. A. 1980. “Egg Shell Quality: II. Effect of Dietary Manipulations of Protein, Amino Acids, Energy, and Calcium in Young Hens on Egg Weight, Shell Weight, Shell Quality, and Egg Production.” Poultry Science 59, no. 9: 2047–2054.

[vms370789-bib-0028] Tepox Pérez, M. D. L. Á. , B. F. Martínez , T. J. Méndez , and E. Á. González . 2012. “Different Levels of Metabolizable Energy and Sulfur Amino Acids in Diets for White Bovans Hens.” Revista Mexicana de Ciencias Pecuarias 3, no. 4: p439.

[vms370789-bib-0029] Wang, H. , X. Wang , J. Zhao , H. Jiao , and H. Lin . 2020. “Low Protein Diet Supplemented With Crystalline Amino Acids Suppressing Appetite and Apo‐Lipoprotein Synthesis in Laying Hens.” Animal Feed Science and Technology 266: 114533.

